# Myocardial Infarction With Non-Obstructive Coronary Artery Disease: Exploration of the Etiology to Guide Therapy

**DOI:** 10.31083/RCM49253

**Published:** 2026-06-29

**Authors:** Angelo Giuseppe Marino, Antonio De Vita, Saverio Tremamunno, Nello Cambise, Fabio De Benedetto, Gaetano Antonio Lanza

**Affiliations:** ^1^Department of Cardiovascular Sciences, Fondazione Policlinico Universitario A. Gemelli IRCCS, 00168 Rome, Italy; ^2^Università Cattolica del Sacro Cuore, 00168 Rome, Italy

**Keywords:** acute coronary syndrome, myocardial infarction, coronary vasospasm, coronary dissection, blood vessel, coronary vessel, coronary thrombosis

## Abstract

Myocardial infarction with non-obstructive coronary artery disease (MINOCA) occurs in up to 14% of patients presenting with acute myocardial infarction. Initially considered a favorable clinical diagnosis, MINOCA is now recognized as a condition that significantly impairs quality of life and is associated with an unfavorable prognosis, including significant risks of mortality, rehospitalization, disability, and recurrent angina, all contributing to high socioeconomic costs. MINOCA is a heterogeneous condition, with multiple identified underlying mechanisms, including epicardial or microvascular spasm, rupture or erosion of an atherosclerotic coronary plaque, coronary embolism, and spontaneous coronary artery dissection. Given this complexity, a comprehensive diagnostic workup that integrates clinical assessment, advanced imaging modalities, and invasive testing is necessary for the accurate identification of the specific cause of MINOCA and to guide the selection of an appropriate, individualized therapeutic strategy for each patient. This narrative review aims to explore the etiologies of MINOCA and provide clinicians with an up-to-date overview of therapeutic advances and targeted strategies for each underlying mechanism.

## 1. Introduction

According to the European Society of Cardiology (ESC), myocardial infarction with non-obstructive coronary artery disease (MINOCA) is defined as the occurrence of an acute myocardial infarction in the absence of obstructive coronary artery disease, as determined by invasive coronary angiography (ICA) demonstrating less than 50% stenosis in any major epicardial vessel. Additionally, the diagnosis requires the exclusion of alternative non-coronary causes capable of mimicking myocardial infarction, such as systemic infections (e.g., sepsis), severe anemia, tachyarrhythmias, or pulmonary embolism [1]. When applying this definition MINOCA has been reported to occur in up to 14% of patients admitted with a clinical presentation of acute myocardial infarction [2].

This precise and stringent definition has important implications for clinical practice, as it effectively excludes several conditions that were previously grouped under the MINOCA umbrella, such as takotsubo syndrome and myocarditis, which are now recognized as separate clinical entities with distinct etiologies and management strategies. The ESC definition underscores the central role of proven myocardial ischemia in the pathogenesis of MINOCA, reinforcing the need for a focused diagnostic approach to identify the underlying ischemic mechanisms leading to myocardial damage.

Although MINOCA was initially perceived as a relatively benign and favorable diagnosis compared to classic myocardial infarction caused by obstructive coronary artery disease [3], this notion has been challenged by accumulating evidence demonstrating that MINOCA is far from being a harmless condition. Patients diagnosed with MINOCA are indeed exposed to a significant risk of adverse clinical outcomes, including increased mortality, recurrent hospitalizations, long-term disability, and persistence or recurrence of anginal symptoms. These clinical sequelae contribute not only to impaired quality of life for affected individuals but also to a considerable socio-economic burden on healthcare systems [4,5,6].

MINOCA is a heterogeneous syndrome, however, and clinical outcome likely depends on the specific pathophysiological mechanism responsible for its occurrence. The spectrum of the causes of MINOCA includes epicardial or microvascular coronary vasospasm, the rupture or erosion of a non-obstructive atherosclerotic plaque, coronary embolism originating from cardiac or extracardiac sources, microvascular thrombosis and spontaneous coronary artery dissection [7,8].

The wide range of potential etiologies makes the diagnosis and management of MINOCA particularly complex, necessitating a comprehensive, stepwise diagnostic workup that integrates clinical assessment, advanced imaging modalities, and sometimes invasive testing. This approach is essential to accurately identify the precise cause in each individual patient, which, in turn, is critical for guiding the selection of the most appropriate and effective therapeutic strategy [9,10,11,12,13,14,15].

In this narrative review, we aim to provide an in-depth exploration of the diverse mechanisms underlying MINOCA, offering clinicians an updated and evidence-based overview of current diagnostic tools, therapeutic advances, and targeted management strategies tailored to the specific etiologies.

To this scope, we reviewed in detail all relevant original articles published on the topic in English language in the medical literature. Articles were identified through targeted searches in PubMed/MEDLINE and Embase using combinations of keywords related to MINOCA and its main underlying mechanisms (e.g., coronary spasm, coronary microvascular spasm, plaque disruption, coronary thrombosis, coronary embolism, coronary artery dissection) and diagnostic tools (cardiac magnetic resonance, intracoronary imaging, provocative testing), as well as relevant guideline/consensus documents.

**Fig. 1.**
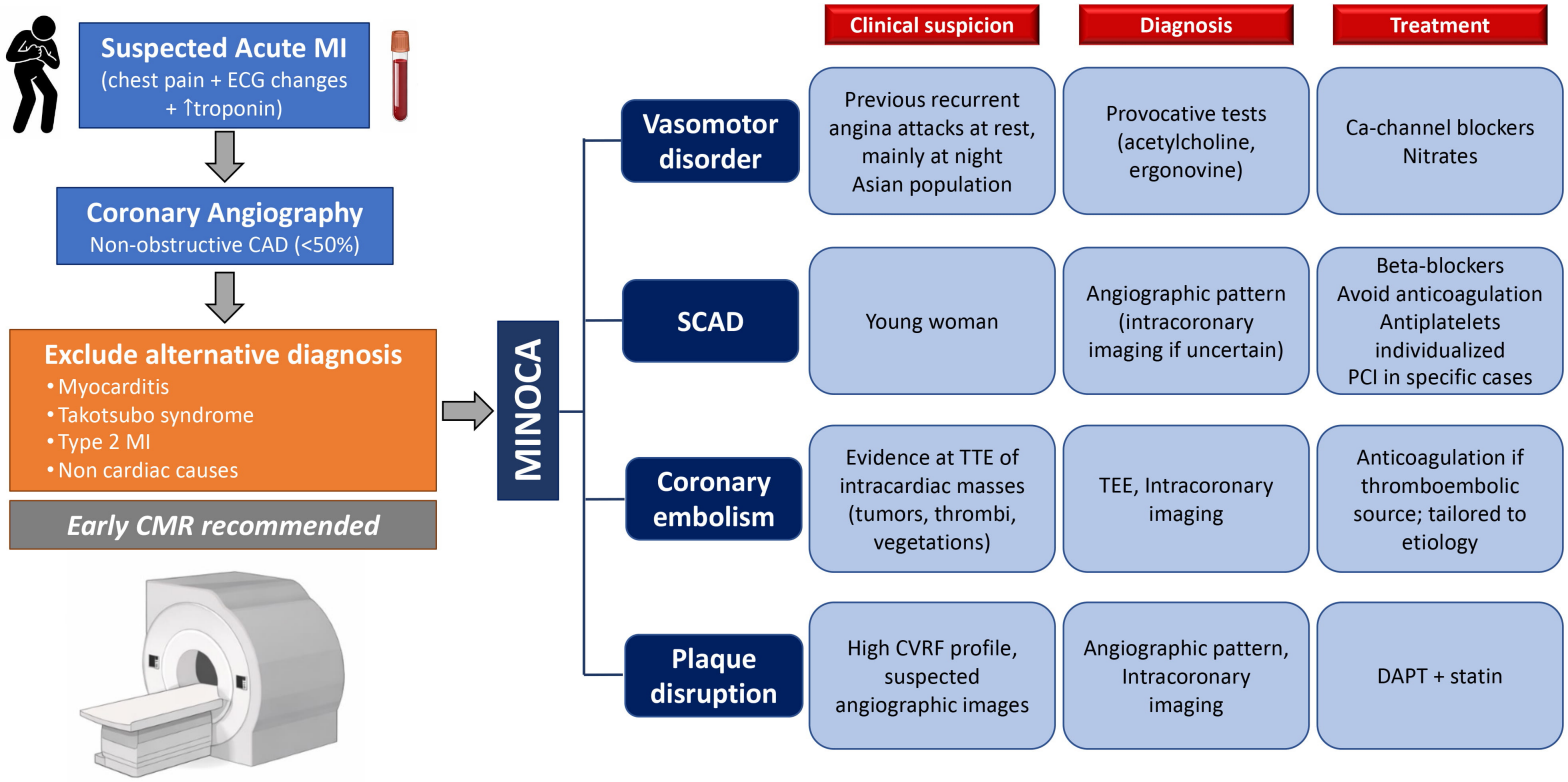
**Diagnostic and therapeutic management of patients with myocardial infarction with non-obstructive coronary artery disease (MINOCA)**. ACEi, angiotensin-converting enzyme inhibitors; CAD, coronary artery disease; CMR, cardiac magnetic resonance; CVRF, cardiovascular risk factors; DAPT, dual antiplatelet therapy; MI, myocardial infarction; PCI, percutaneous coronary intervention; SCAD, spontaneous coronary artery dissection; TEE, transesophageal echocardiogram; TTE, transthoracic echocardiogram.

A summary of the mechanisms and relative tailored diagnostic approach and therapeutic management of patients with acute chest pain and non-obstructive coronary artery disease is shown in the Fig. 1.

As shown, the diagnosis and assessment of MINOCA involves excluding other specific conditions of myocardial injury in the absence of obstructive coronary artery disease. A significant contribution to a correct framework of the clinical picture is given by cardiac magnetic resonance (CMR), which can particularly distinguish between the possible presence of either an ischemic or an inflammatory pattern of myocardial damage. To this scope, CMR is recommended as soon as possible (ideally within three days) when the diagnosis of the acute clinical presentation is doubtful [16].

Once alternative diagnoses have been excluded—most importantly type 2 myocardial infarction, Takotsubo syndrome, and myocarditis (the latter often supported by cardiac magnetic resonance)—the working diagnosis of MINOCA can be established. At that stage, the subsequent diagnostic work-up should not be interpreted as a single, uniform stepwise flowchart; rather, the selection and timing of diagnostic tests should be guided by the initial clinical suspicion and by local availability/expertise.

In patients of Asian ancestry and/or with a history of recurrent angina at rest, particularly with nocturnal episodes, vasospastic angina should be strongly suspected and provocative coronary testing should be considered to confirm the diagnosis. In young women, spontaneous coronary artery dissection (SCAD) should be considered; when angiographic findings are inconclusive, intracoronary imaging may be required to establish the diagnosis. When coronary embolism is suspected, targeted investigations aimed at identifying the embolic source are warranted. Finally, when plaque disruption is suspected despite non-obstructive coronary arteries, intracoronary imaging should be performed to confirm the underlying mechanism.

Importantly, identifying the underlying mechanism of MINOCA has direct therapeutic implications, as it enables an aetiology-guided treatment strategy rather than empiric “one-size-fits-all” therapy. This concept has been recently supported by the PROMISE trial, in which a stratified approach—based on a comprehensive diagnostic assessment followed by tailored, mechanism-specific pharmacological management—resulted in a significant improvement in angina-related health status at 12 months compared with standard care [17].

Accordingly, once a coronary vasomotor disorder is diagnosed, calcium-channel blockers should represent the cornerstone of therapy [18,19,20,21,22,23,24]. In patients with SCAD, a conservative strategy is generally preferred, and percutaneous coronary intervention (PCI) should be reserved for selected cases with haemodynamic or electrical instability, or persistent/recurrent ischemia despite medical therapy [25,26]. When coronary embolism is suspected or confirmed, management should be individualized according to the embolic source and may include anticoagulation when appropriate [27,28,29,30,31,32]. Conversely, when plaque disruption is identified, an antithrombotic strategy (including dual antiplatelet therapy when indicated) and intensive lipid-lowering therapy (including statins) should be implemented in keeping with an atherothrombotic mechanism [29,33].

## 2. Coronary Vasomotor Disorders

Coronary vasomotor disorders, including both epicardial spasm and microvascular spasm, have been recognized to be important mechanisms of MINOCA [9,11,13,27]. Accordingly, once obstructive coronary artery disease has been excluded at ICA, intracoronary provocative testing could be performed to assess whether they are responsible for the syndrome in an individual patient [9].

Various pharmacological agents have been evaluated to induce coronary artery spasm (either epicardial or microvascular) for diagnostic purpose, but acetylcholine and ergonovine are the most widely used in clinical practice [9]. Notably, while all main studies on the assessment of coronary functional abnormalities used invasive coronary functional tests, we have recently given evidence that the presence of an increased coronary vasoconstrictor activity in patients with known non obstructed epicardial coronary arteries can reliably be identified by evaluating the response to constrictor systemic stimuli (ergonovine, hyperventilation) of coronary blood flow velocity in the left anterior descending coronary artery using the simple non-invasive method of transthoracic echocardiographic color-Doppler recording [10,34].

Importantly, identifying coronary vasomotor disorders as the underlying mechanism of MINOCA has direct therapeutic implications, as it enables a targeted, aetiology-guided treatment strategy rather than empiric therapy, as also recently supported by the results of the PROMISE trial [17].

### 2.1 Epicardial Spasm

Epicardial spasm is defined as a transient, intense, focal or diffuse, vasoconstriction of one or more epicardial coronary arteries that causes an occlusion or subocclusion (>90%) of the vessel(s), resulting in a critical reduction of coronary blood flow and consequent myocardial ischemia, thus causing angina and ischemic electrocardiogram (ECG) changes [35,36].

On average, epicardial spasm has been demonstrated by provocative acetylcholine test in about 35% of patients with MINOCA, but its prevalence may depend on whether patients with a preceding history of typical Prinzmetal variant angina (recurrent attacks of rest angina, mainly occurring at night or in the early morning) [9,11,13,35], in whom epicardial spasm is highly probable, are or not included in the diagnosis [9,11]. Furthermore, the prevalence may have significant variations across ethnic groups, being more common in the Asian populations [37]. This ethnic variability may be related to genetic background. Recent genetic studies have indeed suggested that variants at the RNF213 locus may play a role in vasospastic angina, also suggesting a potential contribution to the reported ethnic differences in vasomotor reactivity [38]. However, it should be observed that lifestyle differences, also resulting in differences in cardiovascular risk profile, might also contribute to ethnic differences reported in the occurrence of vasospastic disorders.

#### Treatment

In some patients, a specific trigger of epicardial spasm responsible for MINOCA can be identified, such as cocaine, sympathomimetic and parasympathomimetic drugs, ergot alkaloids, and the chemotherapeutic agent 5-fluorouracil. In these patients, it is essential, and probably sufficient, to avoid exposure to the etiologic agent known to provoke vasospastic episodes in the future [28,35,39,40].

In the majority of patients, however, no specific trigger of spasm can be identified. In these patients, treatment is based on the prescription of calcium channel blockers [18,19], due to the demonstrated high efficacy of these drugs to prevent epicardial spasm, also resulting in a significant reduction of major adverse cardiovascular events (MACE) [19,20,21,22,23,24]. Notably, it has been reported that, in MINOCA patients who tested positive for coronary artery spasm on ACh testing, discontinuation of calcium channel blocker therapy was associated with an increased rate of fatal events [12].

Beta-blockers should be used with caution or avoided in this group of patients, particularly when epicardial spasm has been confirmed by invasive intracoronary provocative testing [28,29,35,40].

In cases of incomplete effects of calcium-channel blocker therapy, some other classes of vasodilator drugs can be added to calcium-antagonist therapy. These may include nitrates and nicorandil, a vasodilator that acts on potassium channels, which can be used as an effective alternative, although its use is often limited by multiple side effects [28,29,35,41].

Furthermore, an effective management of epicardial spasm also requires the correction of some modifiable risk factors that may favor spasm induction, including smoking cessation and a substantial reduction in alcohol consumption [28,35,42].

Possible favorable effects have also been suggested by the adjunctive use of statins, possibly due to their favorable effects on endothelial function and their anti-oxidative and anti-inflammatory effects. Data, however, are controversial and they cannot be routinely recommended in these patients, unless otherwise indicated [43,44].

### 2.2 Microvascular Spasm

Microvascular spasm refers to an abnormal constrictive response of the coronary microcirculation, which, similar to epicardial spasm, leads to an acute impairment of myocardial perfusion [45]. It is diagnosed during invasive provocative testing when typical chest pain and ischemic ECG changes are induced by acetylcholine, but no hemodynamically significant epicardial constriction is shown by coronary angiography [46]. When defined in this way, coronary microvascular spasm has been shown to occur, on average, in approximately 20–30% of patients with MINOCA [11].

At variance with those with epicardial spasm, patients with coronary microvascular spasm are more often female and do not seem to present specific modifiable risk factors [45,47].

Importantly, while recurrence of epicardial spasm can be associated with a sizeable rate of major coronary events, including death and cardiac arrest [35], whether the recurrence of coronary microvascular spasm (CMVS) is associated with a significant rate of major clinical events remains to be demonstrated.

#### Treatment

Similar to epicardial spasm, the prevention of CMVS is based on the prescription of calcium-channel blockers [47,48,49]. Unfortunately, however, several data suggest that the efficacy of these drugs on the prevention of CMVS is not as strong as that on epicardial spasm [50,51,52]. In the recent EDIT-CMD trial, diltiazem failed to improve angina symptoms in patients with angina and non-obstructive coronary artery disease, and this seemed related to a scarce effect on coronary microvascular spasm [53] and in agreement with earlier evidence that, in patients with documented microvascular spasm, angina recurrence or persistence remains common despite treatment with calcium-channel blockers [51,52].

Similar poor effects on the prevention of coronary microvascular spasm are expected by the prescription of other vasodilator drugs, such as nitrates [54]. In patients with persistent symptoms, adjunctive therapies can be considered, such as nicorandil, for which beneficial effects have been reported in patients with microvascular angina [55]. Furthermore, promising effects on CMVS have been reported with the Rho-kinase inhibitor fasudil, but these types of drugs are not available for chronic use at present [56].

Finally, whether beta-blockers should also be avoided in this group of patients, as in those with epicardial spasm, remains unknown. In fact, we recently found no clinically relevant differences in symptomatic outcome of patients with microvascular angina and CMVS treated with metoprolol or diltiazem [50].

## 3. Plaque Rupture or Erosion

The use of intracoronary imaging allows for the identification of pathological alterations responsible for MINOCA that are not detectable by angiography alone, such as certain mechanisms of coronary plaque instability, including plaque rupture or erosion [14,15,57].

Indeed, the disruption of non-obstructive atherosclerotic plaques—whether through erosion, ulceration, or rupture—represents a significant mechanism underlying MINOCA, potentially explaining between 5% and 20% of cases in most studies, but up to 40% in some reports [58,59].

Conventional coronary angiography often allows only a suspicion of such events, typically through the identification of areas of ‘haziness’, defined as regions of blurred radiopacity. However, it is only through intracoronary imaging techniques, such as intravascular ultrasound (IVUS) and optical coherence tomography (OCT), that these types of lesions can be accurately characterized. Compared to IVUS, OCT provides superior resolution for assessing plaque composition [57].

Plaque rupture, as identified by OCT, is characterized by the presence of a discontinuity in the fibrous cap accompanied by the formation of a cavity within a lipid-rich plaque, often associated with thrombotic material visible within the vessel lumen. However, in MINOCA patients, intracoronary imaging frequently reveals the presence of an intraplaque cavity (ulceration) without evidence of thrombosis. This may reflect cases in which effective fibrinolysis has occurred and possible distal embolization into the microcirculation has taken place [60].

Plaque erosion, on the other hand, is diagnosed by OCT based on exclusion criteria relative to rupture. It is often characterized by the presence of ‘white thrombus’—thrombotic material exhibiting low attenuation on OCT infrared imaging—in the absence of visible discontinuities or ruptures of the fibrous cap, although irregularities of the plaque luminal surface may sometimes be observed [60,61].

In MINOCA patients exhibiting subcritical plaque disruption, CMR imaging often detects extensive myocardial edema, which may be accompanied by limited necrotic regions, indicating transient flow impairment in a major coronary artery. Alternatively, CMR might identify a smaller, sharply demarcated area of late gadolinium enhancement (LGE), corresponding to involvement of a smaller vessel and pointing toward embolization of atherothrombotic material from the rupture site as the predominant cause of myocardial injury [58].

In this regard, a comprehensive CMR performed soon after admission—ideally within 3 days—substantially increases the likelihood of establishing a final diagnosis in MINOCA [16]. When CMR is not immediately available, conventional coronary angiography may still provide useful clues to suspect plaque disruption (e.g., angiographic ‘haziness’), thereby supporting an initial working diagnosis and ACS-oriented management while awaiting definitive characterization by intracoronary imaging and/or deferred CMR.

Importantly, confirmation of plaque disruption supports a stratified, mechanism-guided therapeutic approach in MINOCA, in line with the PROMISE trial, which suggested improved symptomatic outcomes with aetiology-guided management compared with empiric treatment [17].

### Treatment

The therapeutic management of these acute plaque-related events, which include plaque rupture, erosion, or ulceration leading to myocardial injury in the context of MINOCA, should be guided by treatment principles similar to those established for the broader category of acute coronary syndromes (ACS). The following recommendations are largely extrapolated from ACS guidelines [29] and are likely to be most applicable when plaque disruption is confirmed by intracoronary imaging.

In particular, the initiation of dual antiplatelet therapy (DAPT) is generally recommended for a duration of twelve months in order to mitigate the risk of subsequent thrombotic events and to promote vascular healing. After the completion of this initial intensive antiplatelet phase, patients are usually transitioned to lifelong single antiplatelet therapy (SAPT), most commonly with aspirin, to ensure continued protection against future cardiovascular incidents.

Moreover, statin therapy should be instituted and maintained over the long term, given their well-documented lipid-lowering effects and their pleiotropic benefits, including plaque stabilization, anti-inflammatory properties, and improvement of endothelial function [33].

Nonetheless, despite these standard recommendations, certain aspects of management in the context of MINOCA related to plaque disruption remain less clearly defined. In particular, the role of pharmacological therapies commonly employed in ACS, such as beta-blockers and agents targeting the renin–angiotensin–aldosterone system (RAAS inhibitors) is not yet well established in this specific subset of patients, due to the limited evidence regarding their efficacy in improving outcomes when significant coronary obstruction is absent [33].

## 4. Coronary Embolism

Coronary embolism (CE) can lead to MINOCA by obstructing distal coronary branches or small resistance vessels within the coronary microcirculation. Coronary embolism is estimated to be responsible for about 3% of all ACS [62].

Small single emboli or multiple microemboli may arise from the lysis of partial, non-occlusive thrombi—either angiographically visible or occult—formed on disrupted, non-significant epicardial plaques, as previously described. Alternatively, coronary embolism can directly originate from thrombi located in the left atrium, left ventricle, or pulmonary veins. Additionally, infective or non-infective endocardial vegetations on the aortic or mitral valves, along with intracardiac tumors, may also contribute to coronary embolism and subsequent MINOCA [63,64,65,66,67,68].

Transthoracic and transesophageal echocardiography represent essential and widely utilized imaging modalities for the identification of potential cardiac sources of embolism, particularly in patients with suspected CE. These non-invasive and semi-invasive techniques allow for the detailed evaluation of cardiac chambers and valves, facilitating the detection of thrombi, vegetations, intracardiac tumors, or other structural abnormalities that may serve as embolic sources. As previously discussed, when coronary embolism is secondary to the disruption of an atherosclerotic coronary plaque, it often exhibits distinctive features on both conventional coronary angiography and intracoronary imaging modalities, such as IVUS or OCT, which can aid in confirming the diagnosis [60].

### Treatment

Currently, no evidence-based standard of care exists for the treatment of coronary embolism. In the acute phase, however, in agreement with international guidelines of acute myocardial infarction [29], anticoagulant therapy is recommended, whereas, for the prevention of recurrences, management should be individualized according to the underlying source of the embolus. Thus, when the embolic material originated from a ruptured or eroded non-obstructive coronary plaque, only dual antiplatelet therapy should be continued (see Section 3), whereas anticoagulation should be the basic treatment of patients with a source of emboli from thrombus localized in the left chambers of the heart [27,28,30,31]. Targeted antimicrobial therapy is required, on the other hand, when the embolic material arises from vegetations of infective endocarditis, which also constitutes an indication for urgent surgery [32].

## 5. Spontaneous Coronary Artery Dissection (SCAD)

A cause of MINOCA that can be identified simply by angiography or through intracoronary imaging techniques is SCAD. SCAD is characterized by the presence of a false lumen within the coronary artery wall, which can compress the true lumen and lead to ischemia [69].

SCAD predominantly affects young women and accounts for approximately up to 4% of all ACS [70].

Angiographically, SCAD is classified into four categories: type 1, characterized by the classic double-lumen appearance; type 2, presenting as a long diffuse narrowing of the vessel lumen greater than 20 mm; type 3, defined by a focal luminal stenosis (length <20 mm); and type 4, in which there is complete occlusion of the vessel. Types 2 and 3 are the most challenging to differentiate from atherosclerotic lesions using angiography alone, but the use of IVUS or OCT usually allow to achieve the correct diagnosis [69,71,72,73].

OCT allows for the visualization of any endothelial rupture site (the so-called ‘intimal tear’) and the resulting intimal flap, as well as the precise distribution and extent of the true and false lumen (the intramural hematoma) [73,74].

IVUS, on the other hand, due to its high tissue penetration, also allows for complete visualization of the intramural hematoma. In patients with suspected SCAD, IVUS can be preferred as it does not require contrast injection, which could potentially extend the dissection and increase the risk of complete vessel lumen occlusion [75].

### Treatment

The preferred therapeutic strategy for SCAD is an initial conservative approach when distal coronary flow is preserved and no high-risk features are present, reserving revascularization (PCI/CABG) for selected patients with ongoing or recurrent ischemia, haemodynamic or electrical instability, or critical proximal/left main involvement. In addition, strict blood pressure control should be emphasized as a key component of long-term management to reduce the risk of recurrence [25,26].

The primary goal of treatment in these patients should focus on preventing the recurrence of SCAD as well as on alleviating the associated symptom of chest pain, which can significantly affect the patient’s quality of life [30]. Consequently, the implementation of antianginal medications is strongly advised, with particular emphasis on the use of beta-blockers, given their proven efficacy in reducing myocardial oxygen demand and potentially lowering the risk of further arterial injury; this therapeutic approach is recommended not only in the acute phase but also as part of long-term management to maintain cardiovascular stability [25,26,76,77,78,79].

The administration of anticoagulant drugs should generally be avoided or discontinued, as their use may increase the likelihood of extension or propagation of the coronary artery dissection, thereby exacerbating the clinical scenario [69,80].

The role of antiplatelet agents in this patient population remains a subject of ongoing debate and uncertainty, with conflicting evidence about their benefit and safety profile. Indeed, on one hand, the dissection itself may exert a prothrombotic effect, both due to the mechanical compression of the true lumen and because the intimal tear may act as a trigger for platelet activation and thrombus formation. On the other hand, antiplatelet therapy could paradoxically facilitate the extension or propagation of the dissection, potentially worsening the structural integrity of the vessel wall [71,81]. In this scenario, intravascular imaging may be helpful in guiding management. In fact, in the presence of an imaging-confirmed intimal tear/intimal flap, antiplatelet therapy may be reasonable to mitigate true-lumen thrombosis; whereas in isolated intramural hematoma without an evident intimal rupture, a less intensive strategy may be preferred given concerns about potential hematoma propagation.

Furthermore, observational data from the DISCO registry reported a higher 12-month MACE rate in patients discharged on DAPT compared with SAPT, suggesting caution with prolonged DAPT and supporting early transition to SAPT when antiplatelet therapy is used in conservatively managed SCAD [82].

Finally, the application of statin therapy in the context of SCAD is supported by only limited and inconclusive data, underscoring the need for further research to establish clear guidelines on their use in this specific clinical setting [83].

## 6. Future Perspective

Despite the growing understanding of the underlying mechanisms of MINOCA, many questions remain unresolved, and future research efforts are crucial to optimizing the management of this heterogeneous condition. One of the main challenges lies in the development of standardized diagnostic algorithms that incorporate multimodality imaging—such as intracoronary imaging and cardiac magnetic resonance—in a structured and widely applicable clinical pathway. This was the goal of the recently published PROMISE trial [17], which has suggested that management of MINOCA patients based on a comprehensive diagnostic assessment and aetiology-guided therapy, may lead to a better symptomatic outcome and health status compared to empiric treatment.

However, further large, prospective, and randomized clinical trials specifically designed for MINOCA patients are needed to establish evidence-based therapeutic strategies tailored to the distinct etiologies underlying the syndrome. Currently, most treatment recommendations are extrapolated from broader ACS populations or based on observational studies, resulting in significant uncertainty in clinical decision-making.

Advanced imaging—potentially supported by AI-driven tools—may facilitate faster and more reproducible differentiation between ischaemic and non-ischaemic presentations and between MINOCA mechanisms, enabling more consistent care pathways.

Emerging fields of investigation include the genetic and molecular characterization of patients predisposed to SCAD, coronary vasomotor disorders or prothrombotic state, which could open the way for personalized preventive medicine approaches [38,84]. Additionally, the role of novel pharmacological agents, revealed to be useful in experimental studies, such as Rho-kinase inhibitors, deserves further exploration and development [56].

Lastly, establishing structured long-term follow-up programs, supported by a multidisciplinary team including cardiologists, radiologists, and geneticists, could improve the comprehensive management of MINOCA patients, enhancing both clinical outcomes and quality of life, while reducing the associated socio-economic costs.

## 7. Conclusion

MINOCA represents a complex clinical entity characterized by a wide spectrum of underlying pathophysiological mechanisms that extend beyond the traditional concept of coronary artery disease. Its heterogeneity requires an appropriate diagnostic workup and individualized therapeutic strategies.

Advances in intracoronary imaging and cardiac magnetic resonance have greatly improved the ability to identify the underlying causes of MINOCA, facilitating more personalized treatment approaches. Nonetheless, clinical practice is still hampered by the lack of specific guidelines and high-quality evidence supporting the efficacy of therapeutic strategies in this patient population.

A deeper understanding of the pathophysiology, coupled with standardized diagnostic pathways and dedicated clinical trials, will be essential to enhance the prognostic stratification and optimize the care of patients with MINOCA.
